# Skin Improvement with Antioxidant Effect of Yuja (*Citrus junos*) Peel Fractions: Wrinkles, Moisturizing, and Whitening

**DOI:** 10.3390/antiox12010051

**Published:** 2022-12-26

**Authors:** Young Yun Jung, In Jin Ha, Mina Lee, Kwang Seok Ahn

**Affiliations:** 1 College of Korean Medicine, Kyung Hee University, 24 Kyungheedae-ro, Dongdaemun-gu, Seoul 02447, Republic of Korea; 2 Korean Medicine Clinical Trial Center (K-CTC), Korean Medicine Hospital, Kyung Hee University, Seoul 02447, Republic of Korea; 3 College of Pharmacy and Research Institute of Life and Pharmaceutical Sciences, Sunchon National University, 255 Jungangno, Suncheon 57922, Republic of Korea

**Keywords:** Yuja (*Citrus junos*), anti-wrinkle, moisturizing, whitening

## Abstract

Yuja (*Citrus junos*) has been cultivated and used for food and medicinal purposes in China and Korea. Its antioxidant, anti-wrinkle, moisturizing, and whitening effects were evaluated in HaCaT, HDF, and B16F10 cells. UVB has been known to cause cellular stress and the production of reactive oxygen species (ROS). Ambivalence of oxidative stress has been reported; however, excessive levels of ROS contribute to skin aging through the loss of elasticity and collagen fibers of connective tissue in the dermis. Skin aging is one of the biological processes that is affected by various factors, including UVB. Pro-Collagen I and hyaluronic acid contents were measured in UVB-irradiated HaCaT and HDF cells to evaluate the anti-wrinkle and moisturizing effects of Yuja-peel (YJP) fractions in -EA (ethyl acetate), -Hex (hexane), and -BuOH (butanol). The expression of matrix metalloproteinases (MMPs) involved in collagen degradation was confirmed to be inhibited by YJP fractions at both the protein and mRNA levels. Filaggrin and serine palmitoyltransferase (SPT), which are moisturizing factors, were induced by YJP fractions. B16F10 cells were treated with α-MSH to induce hyperpigmentation, and then the whitening efficacy of YJP fractions was verified by observing a decrease in melanin content. Overall, our results contribute to the development of various novel skin-improving cosmetics and pharmaceuticals with YJP fractions as active ingredients.

## 1. Introduction

Yuja (*Citrus junos*) is an arboreal species belonging to the genus *Citrus* of Rutaceae. It is found in Sichuan, Hubei, Yunnan, and Tibet in China, and also in Korea [1]. Yuja is mainly prepared in the form of sugar pickles and is used as tea, dressing, and vinegar, and it is widely used for medicinal purposes [1]. Our study confirmed the anti-wrinkle, moisturizing, and whitening effects of the skin using a Yuja-peel (YJP) fraction that has been traditionally used for food and medicine through a molecular mechanism approach.

Skin is a barrier between the organism and the environment [2]. Skin aging reduces its effectiveness as a barrier, and there are intrinsic (cellular metabolism, metabolic processes, and hormones) and extrinsic (pollution, chronic light exposure, chemicals, and toxins) factors that cause aging [2,3]. Specifically, our study focused on skin damage from ultraviolet (UV) radiation. Long-term exposure to ultraviolet (UV) radiation is a major factor of extrinsic skin aging (photoaging), causing wrinkle formation, pigment accumulation, and inflammation reactions [2,4]. According to the wavelength, UV is classified into three types: UVA (320–400 nm), UVB (280–320 nm), and UVC (200–280 nm) [4,5]. UVB (280–320 nm) causes more cellular stress and production of reactive oxygen species (ROS) on human skin than other types of UV cause [4]. UVB radiation can induce collagen degradation and generate inflammatory mediators [6,7].

The ambivalence of oxidative stress has been reported through various previous studies [8,9,10,11]. Excessive levels of reactive oxygen species (ROS) stocks are also known to contribute to tumorigenesis [11]. ROS can be caused by mitochondria and other cells intracellularly; however, it can also be caused by radiation, drugs, tobacco, pollutants, and ultraviolet light externally [12,13,14]. In particular, excessive levels of intracellular ROS lead to the loss of elasticity and collagen fibers of connective tissue in the dermis [15]. An imbalance between glutathione (GSH) and oxidized glutathione (GSSG) by the antioxidant defense system blocks ROS accumulation and prevents skin aging, inflammation, and cancer [16,17].

Matrix metalloproteinases (MMPs) are a family of zinc-containing peptide hydrolases that can lead to the degradation of extracellular matrix proteins (ECMs) [18]. UV-irradiation induces the production of reactive oxygen species (ROS) that can lead to the activation of MMPs, which degrade the collagen matrix system in the dermis [3]. MMP-1 has been reported to lead to collagen degradation due to oxidative stress [6,18].

Dehydration of the skin is closely related to skin aging [19]. Hyaluronic acid modulates hyaluronic acid synthase (HAS-1, 2, and 3) to increase and provide moisture to the skin [20,21]. Filaggrin and serine palmitoyltransferase (SPT) are known to be key factors of hydration [22]. Epidermal barrier protein plays an important role in maintaining skin moisture by forming a protein-lipid matrix, and filaggrin is known as an epidermal barrier protein [21]. SPT has been reported to be related to ceramide biosynthesis among intercellular lipids [23].

Melanin is composed of pigments synthesized in epidermal melanocytes; it prevents and protects the skin from UV rays [24]. However, pigmentation disorders including hyperpigmentation cause various skin diseases, such as freckles, chloasma, and melanoma, due to abnormal melanin production [25,26]. The biosynthesis of melanin can be caused by various stimuli; for example, stimulation by UV-irradiation releases α-melanocyte stimulating hormone (α-MSH) and stimulates melanin biosynthesis in epidermal melanocytes [26]. At this time, α-MSH activates the cAMP-PKA-CREB (cyclic adenosine monophosphate-protein kinase A-cAMP response element binding protein) axis, and the activated cAMP-PKA-CREB axis induces the micropthalmia-associated transcription factor (MITF). MITF lead to increased tyrosinase (TYR), tyrosinase-related protein 1 (TRP-1), and tyrosinase-related protein 2 (TRP-2) in melanocytes stimulated with α-MSH [26,27].

In our study, we demonstrated anti-wrinkle, moisturizing, and whitening effects through molecular pathways using the fractions of Yuja-peel (YJP-EA, -Hex, and BuOH). Our study contributed to expanding the field of the applications of Yuja-peel by proving various effects along with the value of traditional use of citron peel.

## 2. Materials and Methods

### 2.1. Reagents

The seeds of *C. junos*, cultivated in Goheung, Korea, were cold-pressed for the oil extraction. After the removal of the oil, the Yuja-peel (YJP) was extracted with 50% ethanol by sonication for 2 h. The extracted solution was concentrated at room temperature using an evaporator under a speed vacuum. The extract was suspended in distilled H_2_O and partitioned by increasing polarity with *n*-hexane (YJP-Hex), EtOAc (YJP-EA), and *n*-butanol (YJP-BuOH) fractions and water-soluble residue. YJP fractions (YJP-EA, YJP-Hex, and YJP-BuOH) were obtained from the extract and isolated by Dr. In Jin Ha in the Korean Medicine Clinical Trial Center, Kyung Hee University. The fractions were classified into EA (ethyl acetate), Hex (hexane), and BuOH (butanol), according to their chemical properties. 3-(4,5-dimethylthiazol-2-yl)-2,5-diphenyltetrazolium bromide (MTT) and bovine serum albumin (BSA), α-melanocyte stimulating hormone (α-MSH), tyrosinase (from mushroom), and anti-MMP-1 antibody were purchased from Sigma-Aldrich (St. Louis, MO, USA). A Human Pro-Collagen I α1 ELISA kit and a Hyaluronan ELISA kit were purchased from R&D Systems (Minneapolis, MN, USA). Anti-MMP-9, anti-MMP-13, anti-GR, anti-Collagen I, anti-TRP-1, anti-TRP-2, anti-tyrosinase, anti-MITF, and anti-β-actin antibodies were purchased from Santa Cruz Biotechnology (Dallas, TX, USA). Anti-filaggrin and anti-SPT antibodies were obtained from Abcam (Cambridge, UK).

### 2.2. Cell Lines and Culture Conditions

Human keratinocyte HaCaT cells were obtained from Dr. Norbert E. Fusenig (German Cancer Research Center, Heidelberg, Germany). Human dermal fibroblast (HDF) cells and mouse skin melanoma B16F10 cells were obtained from the American Type Culture Collection (Manassas, VA, USA). The HDF cells were cultured in RPMI 1640 and DMEM/F12 medium (1:1) containing 10% feral bovine serum (FBS) and 1% penicillin-streptomycin. The HaCaT and B16F10 cells were cultured in Dulbecco’s Modified Eagle Medium (DMEM) with a low-glucose medium containing 10% feral bovine serum (FBS) and 1% penicillin-streptomycin. The cells’ conditions were maintained at 37 °C in 5% CO_2_.

### 2.3. ROS Production Measurement

The HaCaT cells were irradiated with 30 mJ/cm^2^ of UVB, and the HDF cells were irradiated with 100 mJ/cm^2^. Next, the cells were treated with NAC (3 mM) for 15 min or with YJP-EA, -Hex, and –BuOH (50 μg/mL) for 12 h. The cells were then incubated with cell-permeable fluorescent 2′,7′-dichlorofluorescin diacetate (H2DCF-DA) (10 μM) for 30 min at 37 ℃. Finally, the cells were analyzed using a BD AccuriTM C6 Plus Flow Cytometer (BD Biosciences, Becton-Dickinson, Franklin Lakes, NJ, USA).

### 2.4. Glutathione Measurement

The HaCaT cells were irradiated with 30 mJ/cm^2^ of UVB, and the HDF cells were irradiated with 100 mJ/cm^2^. The cells were then treated with NAC (3 mM) for 15 min or with YJP-EA, -Hex, and –BuOH (50 μg/mL) for 12 h. Next, the supernatants were removed, and the glutathione levels were measured using a GSH/GSSG-Glo^TM^ Assay (Promega, Madison, WI, USA), according to the manufacturer’s protocol [22].

### 2.5. MTT Assay

To evaluate the cell viability of the YJP-EA-, Hex-, and BuOH-treated cells, an MTT assay was used as described previously [28,29]. The cell viability was examined using a VARIOSKAN LUX (Thermo Fisher Scientific Inc., Waltham, MA, USA) at 570 nm.

### 2.6. UVB Irradiation

The HaCaT and HDF cells were seeded in a six-well plate (5 × 10^5^ cells/well). The cells were washed with PBS and subjected to 30 or 100 mJ/cm^2^ of UVB radiation using a CL-1000 Ultraviolet Crosslinker (Ultra-violet products Ltd., Cambridge, UK). After irradiation, the cells were treated with YJP-EA, Hex, and BuOH at the indicated concentrations for 24 h.

### 2.7. Western Blot Analysis

The protein expression levels were evaluated by Western blot analysis using specific antibodies as described previously [29]. The cells (5 × 10^5^ cells/well) were seeded and treated with the various indicated conditions. Whole cell lysates were prepared with equal amounts of proteins, and then the protein expression levels were evaluated by Western blot analysis. The proteins were resolved using a sodium dodecyl sulfate-polyacrylamide gel electrophoresis (SDS-PAGE) and transferred to nitrocellulose membrane. The membrane was then blocked with 5% skimmed milk in 1 × TBST (1 × TBS with 0.1% Tween 20). The proteins were probed by specific antibodies, and the membranes were detected using an enhanced chemiluminescence (ECL) kit (EZ-Western Lumi Femto, DOGEN) (Guro, Seoul, Republic of Korea).

### 2.8. Reverse Transcription PCR

The total RNA was extracted from the cells. The desired RNA was reverse transcribed, and the transcripts were analyzed as indicated in our previous studies [30]. An equal amount of total RNA was reverse transcribed into cDNA, and then RT-PCR was performed to evaluate the expression of MMP-1, -9, -13, and Collagen I. The pairs of forward and reverse primer sets used were as follows: MMP-1, 5′-ATTCTACTGATATCGGGGCTTTGA-3′, and 5′-ATGTCCTTGGGGTGTCCGTGTAG-3′; MMP-9, 5′-TTGAGGAGCGGCTCTCCAAG-3′, and 5′-CGGTCCTGGCAGAAATAGGC-3′; MMP-13, 5′-GGAGCCTCTGAGTCATGGAG-3′, and 5′- TTGAGCTGGACRCATTGTCG-3′; Collagen I, 5′-GGTGGTGGTTATGACTTTGG-3′, and 5′-GTTCTTGGCTGGGATGTTTT-3′. MMP-1 was amplified at 94 °C for 5 min, at 94 °C for 30 s, at 57 °C for 30 s, at 72 °C for 30 s with 27 cycles, and an extension at 72 °C for 7 min. MMP-9 was amplified at 94 °C for 5 min, at 94 °C for 30 s, at 60 °C for 30 s, at 72 °C for 30 s with 30 cycles, and an extension at 72 °C for 7 min. MMP-13 was amplified at 94 °C for 5 min, at 94 °C for 30 s, at 56 °C for 30 s, at 72 °C for 30 s with 27 cycles, and an extension at 72 °C for 7 min. Collagen I was amplified at 94 °C for 5 min, at 94 °C for 30 s, at 50 °C for 30 s, at 72 °C for 30 s with 27 cycles, and an extension at 72 °C for 7 min. Glyceraldehyde-3-phosphate dehydrogenase (GAPDH) was used as the control.

### 2.9. Determination of Pro-Collagen I and Hyaluronic Acid Secretion

The HaCaT and HDF cells were irradiated and treated with YJP-EA, Hex, and BuOH. The culture medium was collected after 24 h of treatment. The Type I Pro-Collagen amount was determined using a Human Pro-Collagen I α1 ELISA kit from R&D Systems (Minneapolis, MN, USA). The hyaluronic acid was determined using a Hyaluronan ELISA kit from R&D Systems (Minneapolis, MN, USA).

### 2.10. Evaluation of Melanin Contents

The B16F10 cells were treated with α-MSH (200 nM) and YJP-EA, Hex, and BuOH for 48 h. The cells were dissolved in 1M NaOH for 2 h at 60 °C and then detected using VARIOSKAN LUX (Thermo Fisher Scientific Inc., Waltham, MA, USA) at 490 nm.

### 2.11. Intracellular Tyrosinase Activity

The B16F10 cells were treated with α-MSH (200 nM) and YJP-EA, Hex, and BuOH for 72 h. The cells were dissolved using a cell lysis buffer, and each lysate was prepared with 20 μL. We then added 80 μL of L-DOPA (2 mg/mL) and incubated the mixtures for 2 h at 37 °C. After 2 h, it was measured using VARIOSKAN LUX (Thermo Fisher Scientific Inc., Waltham, MA, USA) at 490 nm.

### 2.12. Mushroom Tyrosinase Activity

The B16F10 cells were treated with α-MSH (200 nM) and YJP-EA, Hex, and BuOH for 72 h. The supernatants were obtained and prepared with 10 μL. We added 170 μL of L-DOPA (2 mg/mL) and 20 μL of tyrosinase (250 unit) and then incubated the mixtures for 1 h at 37 °C. After 1 h, it was measured using VARIOSKAN LUX (Thermo Fisher Scientific Inc., Waltham, MA, USA) at 475 nm.

### 2.13. Statistical Analysis

All numeric values were represented as the mean ± SD. The statistical significance of the data compared with that of the untreated control was determined using the Student unpaired *t*-test. The significance was set at * *p* < 0.05, ** *p* < 0.01, and *** *p* < 0.001.

## 3. Results

### 3.1. YJP Fractions Inhibit Collagen I Degradation in UVB-Irradiated Human Keratinocyte HaCaT Cells

**Figure 1:**
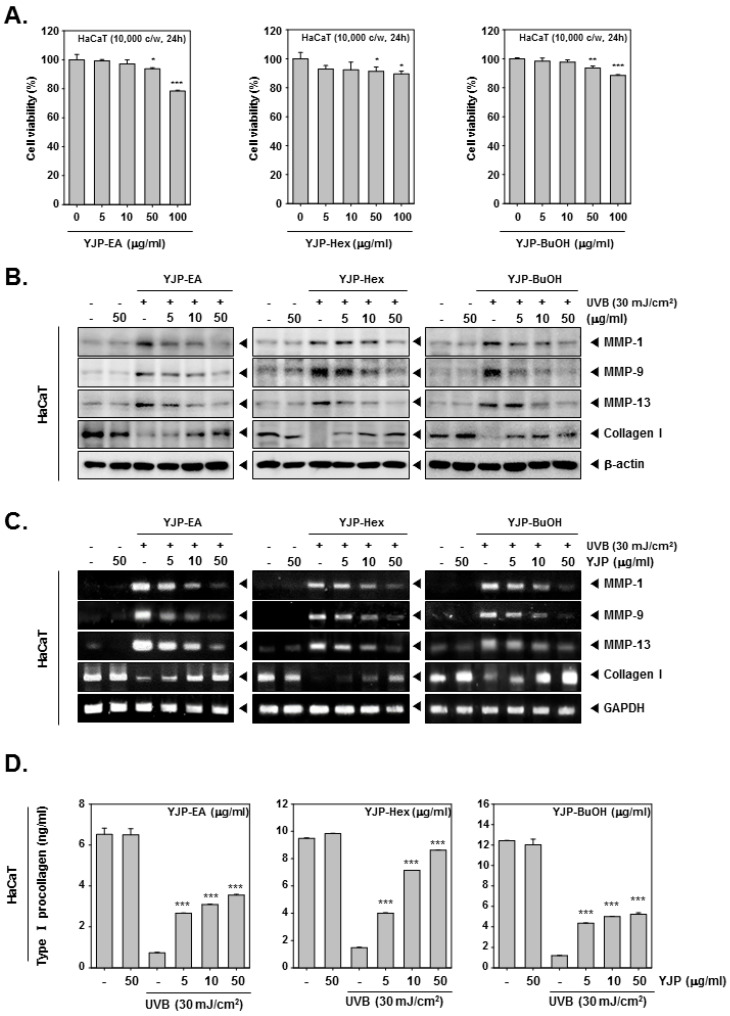
Anti-wrinkle effects of YJP-EA, Hex, and BuOH on UVB-irradiated human keratinocyte HaCaT cells. (**A**) HaCaT cells were treated with YJP-EA, Hex, and BuOH for 24 h. An MTT assay was performed to evaluate cell viability. (**B**) HaCaT cells were irradiated with UVB (30 mJ/cm^2^) and then treated with YJP-EA, Hex, and BuOH for 24 h. Whole cell lysates were analyzed by Western blot analysis. (**C**) HaCaT cells were treated by YJP-EA, Hex, and BuOH for 24 h after UVB (30 mJ/cm^2^) irradiation. The RNA level was evaluated using reverse transcription PCR. (**D**) The Pro-Collagen of HaCaT cells was measured using an ELISA kit, following the manufacturer’s instructions, with 450 nm. All experiments were performed individually in triplicate. *** *p* < 0.001 vs. non-treated (NT) cells, ** *p* < 0.01 vs. non-treated (NT) cells, and * *p* < 0.05 vs. non-treated (NT) cells.

We evaluated the cell viability of HaCaT cells (1 × 10^4^ c/w) under YJP-EA, Hex, and BuOH (0, 5, 10, 50, 100 μg/mL) for 24 h (Figure 1A). At the highest concentration of 100 μg/mL, the cell viability of YJP-EA was the lowest, and all cell viability was near or above 80%. After evaluating the cell viability, the anti-wrinkle effect was confirmed by selecting a concentration with a survival rate above 80%. The HaCaT cells (5 × 10^5^ c/w) were irradiated with UVB (30 mJ/cm^2^) and then treated with YJP-EA, Hex, and BuOH for 24 h. Whole cell lysates were analyzed by Western blot analysis and probed with MMP-1, -9, -13, and Collagen I (Figure 1B). Collagen degradation-related MMP-1, -9, and -13 were increased according to UVB irradiation; however, their expressions were decreased under YJP-EA, Hex, and BuOH treatments. The expression level of Collagen I was suppressed by UVB irradiation, while YJP-EA, Hex, and BuOH increased Collagen I expression. Under the same conditions, to investigate the RNA-level expression, we extracted the RNA from the cells and synthesized cDNA. Using cDNA, we performed a PCR on MMP-1, -9, -13, and Collagen I and then separated it on 1% agar gel (Figure 1C). Similar to that of the protein expression, MMP-1, -9, and -13 were induced by UVB irradiation and suppressed by YJP fractions. The RNA expression of Collagen I was suppressed by UVB irradiation; however, YJP fractions recovered it. Next, the type I Pro-Collagen was measured using the Human Pro-Collagen I α1 ELISA kit. The cells were irradiated with UVB (30 mJ/cm^2^) first and then treated with YJP-EA, Hex, and BuOH for 24 h. As shown in Figure 1D, the type I Pro-Collagen was suppressed by UVB irradiation. However, YJP-EA, Hex, and BuOH recovered the Pro-Collagen content in HaCaT cells. The results show that YJP fractions have anti-wrinkle effects on UVB-irradiated HaCaT cells.

### 3.2. YJP Fractions Inhibit Collagen I Degradation in UVB-Irradiated Human Dermal Fibroblasts (HDF) Cells

**Figure 2:**
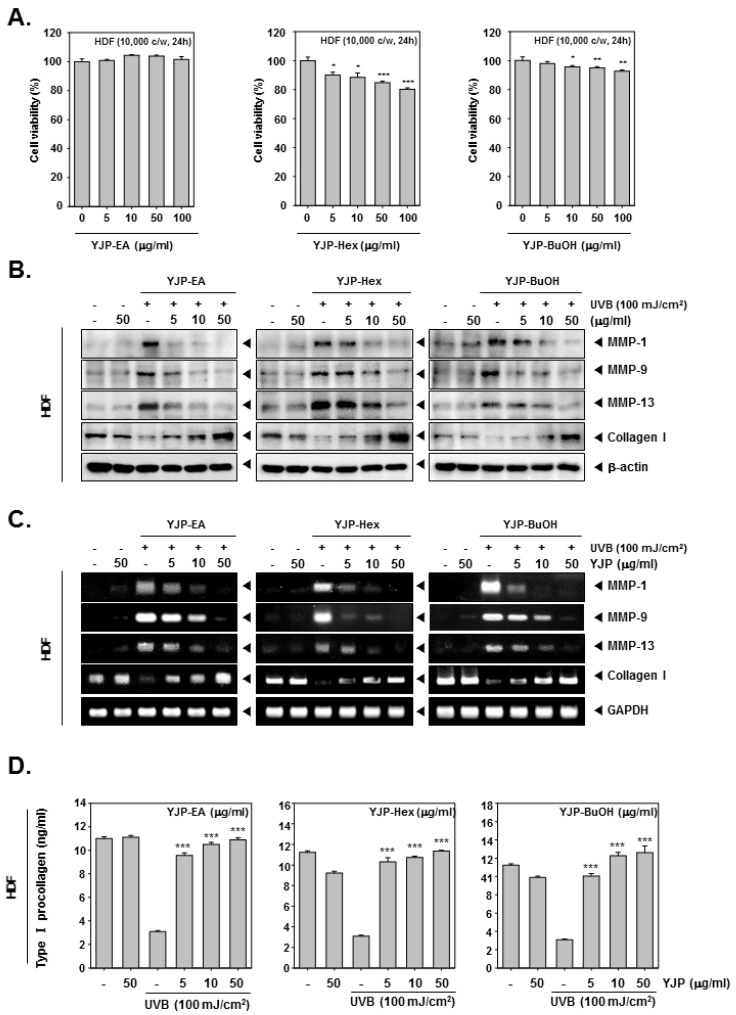
Anti-wrinkle effects of YJP-EA, Hex, and BuOH on UVB-irradiated human dermal fibroblast cells. (**A**) HDF cells were treated with YJP-EA, Hex, and BuOH for 24 h. An MTT assay was performed to evaluate cell viability. (**B**) HDF cells were irradiated with UVB (100 mJ/cm^2^) and then treated with YJP-EA, Hex, and BuOH for 24 h. Whole cell lysates were analyzed by Western blot analysis. (**C**) HDF cells were treated by YJP-EA, Hex, and BuOH for 24 h after UVB (100 mJ/cm^2^) irradiation. The RNA level was evaluated using reverse transcription PCR. (**D**) The Pro-Collagen of HDF cells was measured using an ELISA kit, following the manufacturer’s instructions, with 450 nm. All experiments were performed individually in triplicate. *** *p* < 0.001 vs. non-treated (NT) cells, ** *p* < 0.01 vs. non-treated (NT) cells, and * *p* < 0.05 vs. non-treated (NT) cells.

Next, we investigated the anti-wrinkle effects of YJP fractions on human dermal fibroblasts (HDF) cells. To evaluate cell viability, the HDF cells were treated with YJP-EA, Hex, and BuOH for 24 h, and an MTT assay was examined (Figure 2A). Specifically, YJP-Hex-treated HDF cells showed the lowest cell viability among the YJP fractions. After the MTT assay, we selected the concentrations with a survival rate above 80%. Next, the HDF cells were irradiated with UVB (100 mJ/cm^2^) and treated with YJP-EA, Hex, and BuOH (0, 5, 10, 50 μg/mL) for 24 h. The MMP-1, -9, -13, and Collagen I were analyzed by Western blot analysis with whole cell lysates (Figure 2B). MMP-1, -9, and 13 were induced under UVB irradiation and then suppressed by YJP-EA, Hex, and BuOH. In addition, the YJP fractions restored the expression of collagen, which had been suppressed by UVB. Next, the RNA levels of MMP-1, -9, -13, and Collagen I were investigated using reverse transcription PCR. After the PCR was done, the cDNA was synthesized from RNA, separated on 1% agar gel, and evaluated (Figure 2C). Similar to that of the protein’s expression level, the RNA expressions of MMP-1, -9, and 13 were induced under UVB irradiation and suppressed by YJP-EA, Hex, and BuOH. Collagen I, however, was suppressed by UVB irradiation and recovered by YJP-EA, Hex, and BuOH. The contents of type I Pro-Collagen were decreased with UVB irradiation, and then YJP fractions increased the contents of Pro-Collagen in HDF cells (Figure 2D). The results show that the YJP fractions also have anti-wrinkle effects on UVB-irradiated HDF cells.

### 3.3. YJP Fractions Restore Moisturizing Effects on HaCaT and HDF Cells under UVB-Irradiation

**Figure 3:**
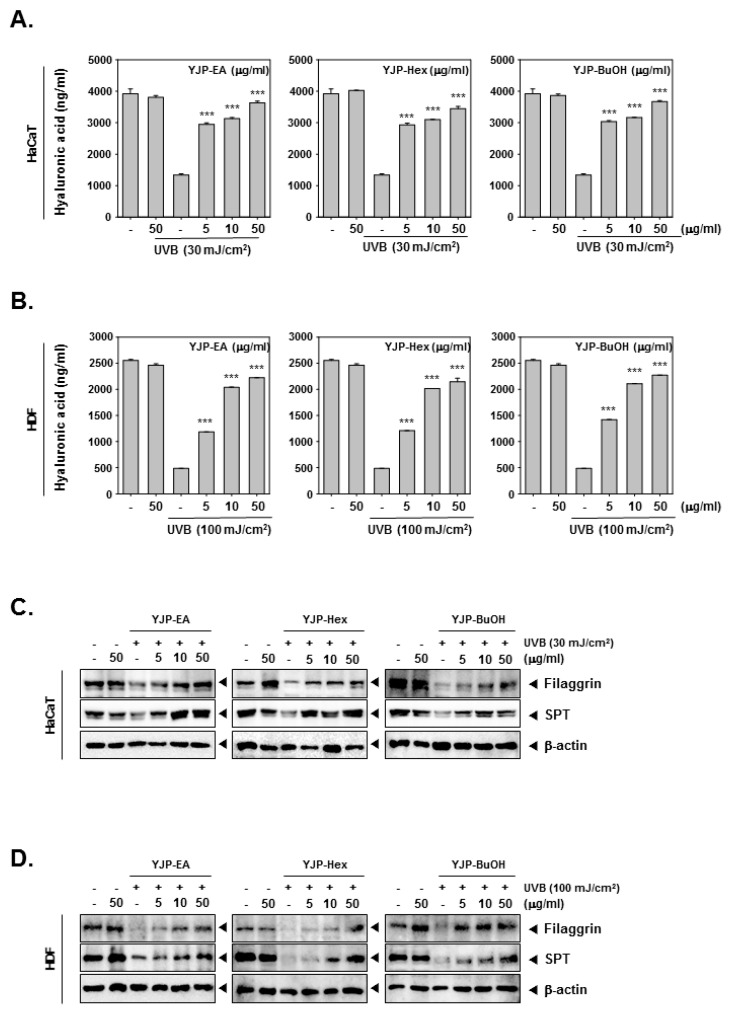
Moisture-recovery effects of YJP-EA, Hex, and BuOH on UVB-damaged HaCaT and HDF cells. (**A**,**B**) HaCaT and HDF cells were irradiated with UVB (30 or 100 mJ/cm^2^) and treated with YJP-EA, Hex, and BuOH for 24 h. The hyaluronic acid was measured using a hyaluronan ELISA kit and detected with 450 nm. (**C**,**D**) The protein-expression levels of filaggrin and SPT were analyzed by Western blot analysis. All experiments were performed individually in triplicate. *** *p* < 0.001 vs. non-treated (NT) cells.

To investigate the moisturizing effects of YJP-EA, Hex, and BuOH, we stimulated HaCaT and HDF cells with UVB irradiation. After UVB irradiation, YJP-EA, Hex, and BuOH (0, 5, 10, 50 μg/mL) were treated for 24 h. The contents of the hyaluronic acid were measured using a Hyaluronan ELISA kit. As shown in Figure 3A,B, the contents of the hyaluronic acid were decreased with UVB irradiation in both HaCaT and HDF cells. However, the treatment of the YJP fraction restored the decreased hyaluronic acid with increasing concentration. Next, we investigated the protein expression of filaggrin and SPT by Western blot analysis to study the molecular mechanism of the moisturizing effect (Figure 3C,D). In both HaCaT and HDF cells, UVB irradiation inhibited the filaggrin and SPT expression. Despite the UVB irradiation, filaggrin and SPT were restored by YJP-EA, Hex, and BuOH. The results show that YJP fractions can recover moisturizing effects in UVB-irradiated HaCaT and HDF cells.

### 3.4. YJP Fractions Suppress Melanin Contents in Mouse Skin Melanoma B16F10 Cells

**Figure 4:**
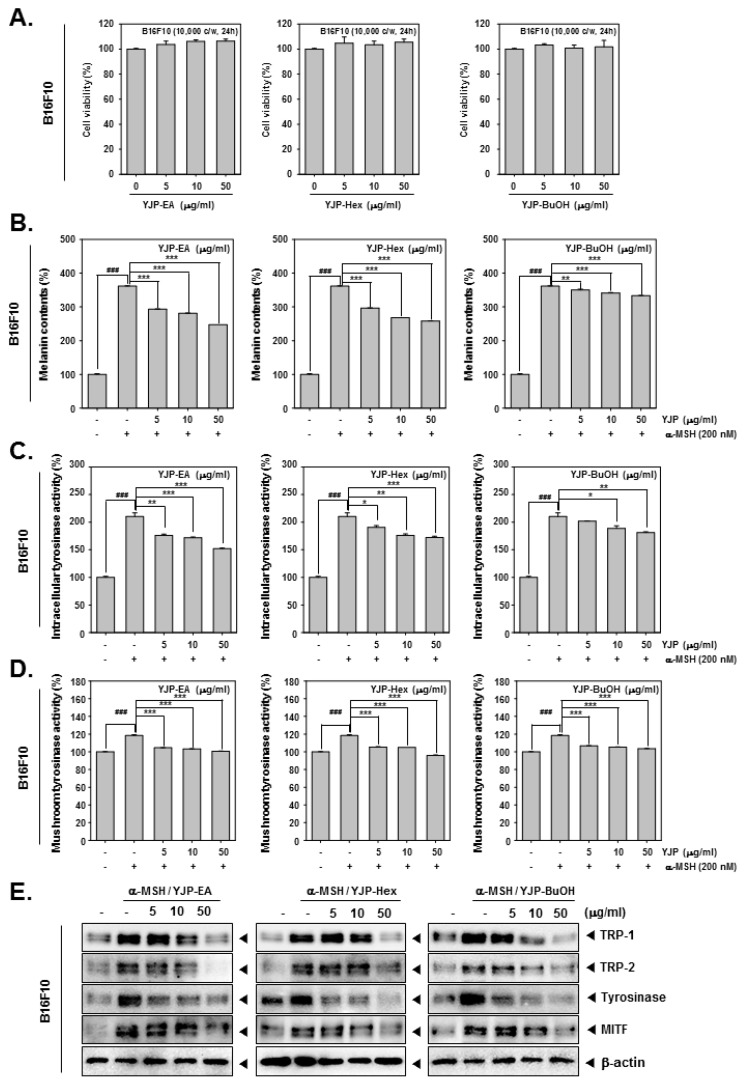
Melanin inhibition effects of YJP-EA, Hex, and BuOH on B16F10 cells. (**A**) B16F10 cells were treated with YJP-EA, Hex, and BuOH for 24 h. Next, the cell viability was measured using the MTT assay. (**B**) Melanin contents from α-MSH-stimulated B16F10 cell. The cells were treated with α-MSH (200 nM) and YJP-EA, Hex, and BuOH for 48 h. The cell lysates were measured at 490 nm. (**C**) To measure intracellular tyrosinase activity, the B16F10 cells were treated with -MSH (200 nM) and YJP-EA, Hex, and BuOH for 72 h. L-DOPA was added into the lysates and measured at 490 nm. (**D**) The mushroom tyrosinase activity was evaluated at -MSH (200 nM) and in YJP-EA-, Hex-, and BuOH-treated B16F10 cells. The supernatants with L-DOPA and tyrosinase were measured at 475 nm. (**E**) The whole cell lysates were analyzed by Western blot analysis. All experiments were performed individually in triplicate. *** *p* < 0.001 vs. α-MSH-treated cells, ** *p* < 0.01 vs. α-MSH-treated cells, and * *p* < 0.05 vs. α-MSH-treated cells. ^###^ *p* < 0.001 vs. non-treated (NT) cells.

We first confirmed the cytotoxicity of YJP-EA, Hex, and BuOH on B16F10 cells using the MTT assay (Figure 4A). We confirmed that all YJP fractions had no cytotoxicity on B16F10 cells. Next, we measured the melanin contents from B16F10 cells treated with α-MSH (200 nM) and YJP-EA, Hex, and BuOH (0, 5, 10, 50 μg/mL) for 48 h. The cell lysates were measured using VARIOSKAN LUX (Thermo Fisher Scientific Inc., Waltham, MA, USA) at 490 nm. The melanin contents were increased by α-MSH, which is known to stimulate melanin synthesis, and then inhibited by YJP fractions. Next, the intracellular tyrosinase activity and mushroom tyrosinase activity were analyzed under the same conditions as that of the B16F10 cells (Figure 4C,D). α-MSH also induced both intracellular tyrosinase activity and mushroom tyrosinase activity. However, the YJP fractions significantly inhibited both activities in the B16F10 cells. From the whole cell lysate, we confirmed the protein-expression levels of TRP-1, -2, and tyrosinase and the microphthalmia-associated transcription factor (MITF), known as the enzymes and factors that promote melanin formation (Figure 4E). TRP-1, -2, tyrosinase, and MITF were induced by α-MSH; however, all YJP fractions reduced it. The results show that YJP-EA, Hex, and BuOH inhibit melanin contents and the related enzyme in mouse skin melanoma B16F10 cells.

### 3.5. YJP Fractions Induce GSH/GSSG Imbalance and Inhibit ROS Production in HaCaT, HDF, and B16F10 Cells

**Figure 5:**
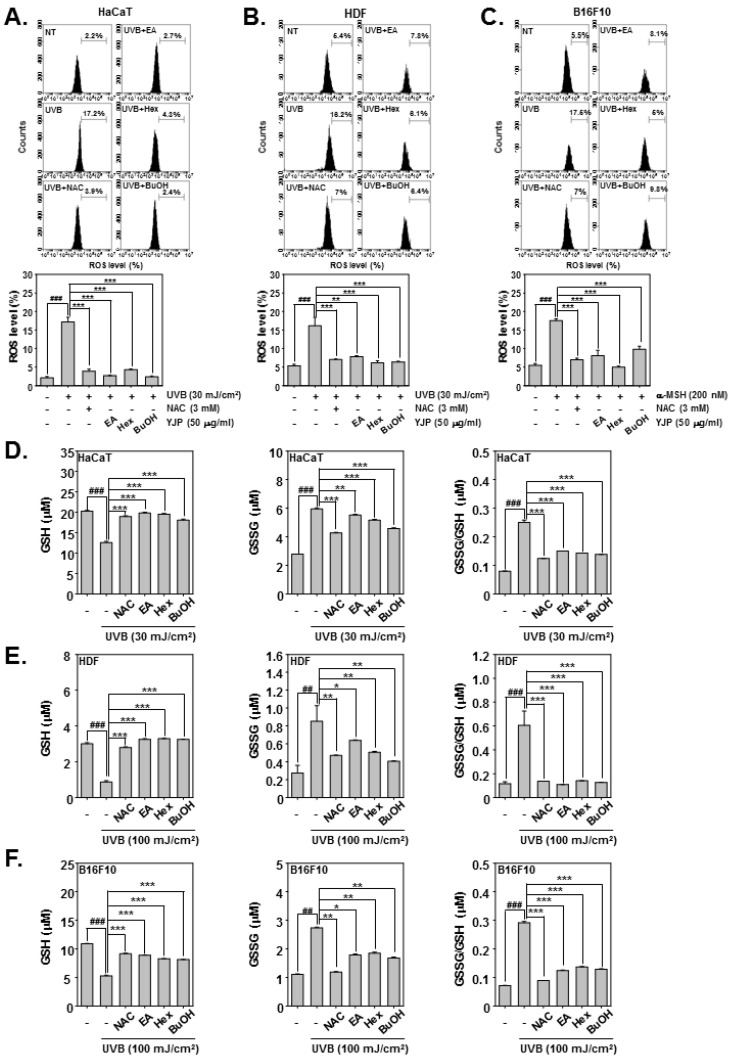
Antioxidant effects of YJP-EA, -Hex, and -BuOH on HaCaT, HDF, and B16F10 cells. HaCaT and HDF cells were irradiated with UVB; B16F10 cells were stimulated by α-MSH. We then treated them with NAC (3 mM) for 15 min or with YJP-EA, -Hex, and -BuOH (50 μg/mL) for 12 h. (**A**–**C**) The ROS production was analyzed by flow cytometer. (**D**–**F**) The GSH and GSSG levels were measured, and the GSSG/GSH ratio was evaluated in both cells. All experiments were performed individually in triplicate. ^###^ *p* < 0.001 vs. non-treated (NT) cells, ^##^ *p* < 0.01 vs. non-treated (NT) cells, *** *p* < 0.001 vs. UVB or α-MSH-stimulated cells, ** *p* < 0.01 vs. UVB or α-MSH-stimulated cells, and * *p* < 0.05 vs. UVB or α-MSH-stimulated cells.

Next, we investigated the antioxidant effects of YJP-EA, -Hex, and -BuOH in HaCaT, HDF, and B16F10 cells under UVB irradiation or α-MSH stimulation. In HaCaT and HDF cells, the ROS production and GSH/GSSG levels were measured with UVB-irradiated cells. The B16F10 cells were stimulated by α-MSH. N-acetyl-l-cysteine (NAC) has been proven to prevent the development of oxidative stress and activate antioxidant enzymes [31]. We used NAC as a positive control. The HaCaT cells were irradiated with 30 mJ/cm^2^ of UVB; the HDF cells were irradiated with 100 mJ/cm^2^; and the B16F10 cells were stimulated with α-MSH (200 nM). The cells were then treated with NAC (3 mM) for 15 min or with YJP-EA, -Hex, and -BuOH (50 μg/mL) for 12 h. First, we evaluated the ROS production in HaCaT, HDF, and B16F10 cells. The cells were incubated with H_2_DCF-DA and analyzed using a flow cytometer. Both UVB irradiation and α-MSH increased the ROS level in HaCaT, HDF, and B16F10 cells. However, YJP fractions reduced the increased-ROS production level (Figure 5A–C). Next, we evaluated the GSH and GSSG levels. The cells were treated the same as when the ROS production was measured. As shown in the results, in HaCaT, HDF, and B16F10 cells, both UVB irradiation and α-MSH reduced the GSH level and increased the GSSG level. As a result, it was confirmed that the GSSG/GSH ratio was increased by UVB irradiation and α-MSH. However, when the YJP fractions were treated, it was confirmed that the decreased GSH level recovered and the GSSG level decreased, despite UVB irradiation and α-MSH (Figure 5D–F).

## 4. Discussion

In our study, we analyzed and evaluated the anti-wrinkle, moisturizing, and whitening effects of Yuja-peel fractions (YJP-EA, -Hex, and -BuOH) in HaCaT, HDF, and B16F10 cells. Prior to the experiment, we first evaluated the toxicity of the three fractions on each cell, and we then conducted a sub-experiment by selecting a concentration with low toxicity. In addition, we stimulated HaCaT and HDF cells with UVB irradiation and B16F10 cells with α-MSH to create a hyperpigmented state [4,26,27].

To evaluate the antioxidant effects, we measured GSH and GSSG levels [16,17]. Under UVB irradiation, cells lost their antioxidant capacity. The ROS productions were increased by UVB; however, the YJP fractions reduced the ROS level in both HaCaT and HDF cells. In addition, the GSH level was reduced and GSSG level was increased by UVB irradiation. However, YJP-EA, -Hex, and –BuOH recovered the GSH level and reduced the GSSG level with N-acetyl-l-cysteine (NAC) [31]. These results demonstrate that YJP fractions have antioxidant effects on HaCaT and HDF cells.

The expression of MMPs (MMP-1, -9, -13), contributing to collagen degradation, were increased in UVB-irradiated HaCaT and HDF cells, while Collagen I was decreased [3,6,18]. However, YJP-EA, -Hex, and -BuOH inhibited MMP-1, -9, and -13 but restored Collagen I in the protein and mRNA levels. In addition, when the Pro-Collagen content was investigated, it was confirmed that the percentage of Type I Pro-Collagen, which had been reduced by UVB-irradiation, was restored by treatment with YJP-EA, -Hex, and -BuOH. These results demonstrate that YJP fractions have significant anti-wrinkle effects through regulating MMPs at low toxicity in both HaCaT and HDF cells.

Next, to confirm the moisturizing effect, we irradiated HaCaT and HDF cells with UVB [19]. We confirmed that the intracellular hyaluronic acid content from both cells were reduced by UVB irradiation and that the treatment of YJP fractions significantly increased the hyaluronic acid. To confirm this moisturizing effect, the protein expressions of filaggrin and SPT, which are key factors of hydration, were evaluated [21,22,23]. Both filaggrin and SPT were suppressed under UVB irradiation; however, the YJP fractions induced their expression. These results show that YJP fractions can recover moisturization through regulating filaggrin and SPT in HaCaT and HDF cells.

In the B16F10 cells, we treated α-MSH to induce hyperpigmentation [25,26]. α-MSH induced hyperpigmentation, and as a result, it was confirmed that melanin content, intracellular tyrosinase activity, and mushroom tyrosinase activity were all increased in B16F10 cells [25,26]. Increased melanin content, intracellular tyrosinase activity, and mushroom tyrosinase activity were decreased by YJP-EA, -Hex, and -BuOH. Additionally, α-MSH-induced MITF, tyrosinase (TYR), tyrosinase-related protein 1 (TRP-1), and tyrosinase-related protein 2 (TRP-2) were all confirmed to have decreased protein expression levels after treatment with YJP fractions [26,27]. These results demonstrate that YJP fractions suppress hyperpigmentation through the modulation of MITF, TRP-1, TRP-2, and tyrosinase.

## 5. Conclusions

Our study demonstrated the potential effects of YJP fractions by confirming their anti-wrinkle, moisturizing, and whitening effects at low toxicity. Depending on the polarity and previous reports [32,33], it was assumed that YJP-BuOH includes flavonoid glycosides, YJP-EA contains limonoids and coumarins, and YJP-Hex is comprised of limonoids and some fatty acids. In particular, the yield of YJP-Hex from *C. junos* seed shells appears to be higher than that of other fractions, and thus there may be some economic advantages associated with product development. These results encourage a focus on the excellence of materials coming from nature.

## Data Availability

The data presented in this study are available on request from the corresponding author.
